# Muc2 mucin o-glycosylation interacts with enteropathogenic *Escherichia coli* to influence the development of ulcerative colitis based on the NF-kB signaling pathway

**DOI:** 10.1186/s12967-023-04687-2

**Published:** 2023-11-08

**Authors:** Juan Wei, Chunyan Chen, Jing Feng, Shuping Zhou, Xiaoyue Feng, Zhao Yang, Heng Lu, Hui Tao, Liuying Li, Huabing Xv, Ji Xuan, Fangyu Wang

**Affiliations:** 1 Department of Gastroenterology and Hepatology, Jinling Hospital, Affiliated Hospital of Medical School, Nanjing University, No. 305 East Zhongshan Road, Nanjing, 210002 People’s Republic of China; 2 Department of Gastroenterology and Hepatology, The First School of Clinical Medicine, Southern Medical University, Guangzhou, 510515 People’s Republic of China; 3 Department of Gastroenterology and Hepatology, Huainan First People’s Hospital and, First Affiliated Hospital of The Medical College of Anhui, University of Science and Technology, Huainan, 232000 Anhui People’s Republic of China; 4 Department of Gastroenterology and Hepatology, Jinling Clinical College of Nanjing Medical University, Nanjing, 210002 People’s Republic of China

**Keywords:** Ulcerative colitis, Intestinal epithelium, o-glycan, *Escherichia coli*, Intestinal epithelial barrier, Gut microbiota

## Abstract

**Background:**

Ulcerative colitis (UC) is a chronic inflammatory disease of the intestine characterized by a compromised intestinal epithelial barrier. Mucin glycans are crucial in preserving barrier function during bacterial infections, although the underlying mechanisms remain largely unexplored.

**Methods:**

A cohort comprising 15 patients diagnosed with UC and 15 healthy individuals was recruited. Stool samples were collected to perform 16S rRNA gene sequencing, while biopsy samples were subjected to nanocapillary liquid chromatography-tandem mass spectrometry (nanoLC-MS/MS) to assess O-glycosylation. Gene expression was evaluated through qPCR analysis and Western blotting. Furthermore, animal experiments were conducted to investigate the effects of *Escherichia coli* and/or O-glycan inhibitor benzyl-α-GalNAc on the development of colitis in mice.

**Results:**

Our findings revealed that the mucus barrier was disrupted during the early stages of UC, while the MUC2 protein content remained unaltered. Additionally, a noteworthy reduction in the o-glycosylation of MUC2 was observed, along with significant changes in the intestinal microbiota during the early stages of UC. These changes included a decrease in intestinal species richness and an increase in the abundance of *Escherichia coli* (*E. coli*). Moreover, subsequent to the administration of galactose or o-glycan inhibitor to intestinal epithelial cells, it was observed that the cell culture supernatant had the ability to modify the proliferation and adhesive capacity of *E. coli*. Furthermore, when pathogenic *E. coli* or commensal *E. coli* were cocultured with intestinal epithelium, both strains elicited activation of the NF-KB signaling pathway in epithelial cells and facilitated the expression of serine protease in comparison to the untreated control. Consistently, the inhibition of o-glycans has been observed to enhance the pathogenicity of *E. coli* in vivo. Furthermore, a correlation has been established between the level of o-glycans and the development of ulcerative colitis. Specifically, a reduction in the O-glycan content of MUC2 cells has been found to increase the virulence of *E. coli*, thereby compromising the integrity of the intestinal epithelial barrier.

**Conclusions:**

Together, there exist complex interactions between the intestinal epithelium, o-glycans, and the intestinal microbiota, which may inform the development of novel therapeutic strategies for the treatment of ulcerative colitis.

**Supplementary Information:**

The online version contains supplementary material available at 10.1186/s12967-023-04687-2.

## Background

Inflammatory bowel diseases (IBD), such as Crohn disease (CD) and ulcerative colitis (UC), are chronic, nonspecific inflammatory conditions that primarily affect the colon and rectum [1]. The incidence of UC is notably high in developed countries and has experienced a rapid increase in newly industrialized nations [2, 3]. Within China, the prevalence of ulcerative colitis is approximately 11.6 per 100,000 individuals [4]. The pathogenesis of this disease is multifaceted and is currently understood to be influenced by genetic factors, host immune system dysregulation, gut microbiota dysbiosis, and environmental factors [5, 6].

Patients diagnosed with UC typically necessitate personalized and thorough treatment [7]. Mild UC cases are commonly addressed with aminosalicylate inhibitors, whereas moderate and severe UC cases necessitate supplementary treatment with corticosteroids, immunosuppressive agents, and biologics [8]. In instances of acute illness, intravenous glucocorticoids are administered to patients after appropriate fluid and electrolyte replacement [8, 9]. In cases of severe UC that are complicated by hemorrhage, perforation, and/or ineffective medical treatment, surgical intervention may be required as necessary [10, 11]. Given that the majority of extant treatment options for UC are symptomatic in nature, patients are required to undergo long-term medication. Discontinuation of medication is prone to result in relapse and the development of drug resistance [12, 13]. Consequently, it is imperative to undertake further investigation into the pathogenesis of UC and to explore potential treatment strategies, with the aim of enhancing the quality of life of affected patients.

The intestinal mucus barrier serves as the primary defense mechanism of the host against environmental, physiological, and immune stimuli, effectively obstructing direct contact between epithelial cells and a range of particles, toxins, pathogens, and commensal bacteria [14, 15]. Previous studies have demonstrated that a compromised mucus barrier is a significant manifestation of UC [16, 17]. The colonic mucus layer represents the primary ecological niche for commensal bacteria, with the composition of the intestinal microbiota being relatively species-specific and regulated by the intestinal mucus [17]. The composition of gut microbiota in individuals with IBD is distinct from that of healthy individuals, and is influenced by both environmental and host-related factors, leading to a disruption of intestinal homeostasis. This disturbance can result in metabolic disorders that may contribute significantly to the pathogenesis of IBD [18, 19].

Mucins constitute the primary constituents of the colonic mucus layer, and the mucin glycans are crucial in preserving barrier function during bacterial and parasitic infections [14, 20]. Mucin-type O-glycosylation has been demonstrated to be intricately linked to mucin function [21, 22]. The suppression of mouse glycosyltransferases resulted in a marked decrease in o-glycan content, which led to intestinal inflammation and the emergence of colorectal tumors in mice [23–25]. The current investigation provides evidence that MUC2 protein expression remains unchanged during the early stages of human UC development, while there is a notable decrease in mucin-type o-glycan biosynthesis. This alteration is linked to the mild clinical symptoms observed in UC patients. However, additional research is required to elucidate the precise mechanism by which O-glycan reduction affects UC pathogenesis.

## Methods

### Patients

The present study involved the collection of UC specimens and paired non-inflammatory colon tissue for pathological observation from patients with UC who underwent colonoscopy at Jinling Hospital. Prior to participation, written informed consent was obtained from all patients, and the study was approved by the Institutional Review Board of Jinling Hospital. A total of 15 patients with UC were included in the study, and sigmoid colon biopsy specimens were evaluated to determine disease activity based on the Mayo Endoscopic Subscore (MES). The clinical characteristics of all patients are presented in Table 1. The inclusion criteria encompass the age range of 18 to 60 years, a diagnosis that has been confirmed by physicians through patient history, colonoscopy, pathology, and imaging, as well as a diagnosis and activity assessment that aligns with the “Consensus opinion on the diagnosis and treatment of inflammatory bowel disease in China” with a Mayo score of ≤ 10. The exclusion criteria encompass individuals who fail to meet the diagnostic and inclusion criteria, those with coexisting severe liver, heart, lung, kidney, and other systemic illnesses, women with a recent childbirth plan or pregnancy, psychiatric patients or uncooperative patients, individuals who have utilized antibiotics and probiotics within the past three months, and patients who have participated in other clinical studies. The healthy control group underwent colonoscopy monitoring to assess colon polyps and presented with no underlying pathology. Additionally, a cohort of 12 patients with UC and 12 healthy individuals provided stool samples for 16S rRNA gene sequencing.

**Table 1:** Clinical characteristics of patients with mild UC

Clinical characteristics	UC (15 cases)	Healthy subjects (15 cases)
Age	mean age = 31 years old (range from 19 to 42)	mean age = 30 years old (range from 24 to 45)
Gender	7 females/8 males	8 females /7 males
Course of disease	All ≥ 6 months	NA
Medication		NA
Salicylic acid	15 cases	–
Hormone	8 cases	–
Biological agents	1 case	–
Enema with traditional Chinese medicine	10 cases	–
Mayo score	Mean value = 7 (rang from 4 to 10)	NA
MES score	Mean value = 2 (rang from 1 to 2)	NA

*UC* ulcerative colitis; *NA* not applicable; *MES* Mayo endoscopic score

### Immunohistochemistry and HE staining

Biopsy specimens were fixed in formalin and embedded in paraffin, and 5 μm thick sections were prepared using standard procedures. The slides were subjected to dewaxing and rehydration, followed by incubation with 3% H2O2 in PBS for 10 min at room temperature. After washing with PBS five times, antigen retrieval was carried out using Citrate-EDTA Antigen Retrieval Solution (Beyotime Biotechnology, #P0068). The sections were then blocked with QuickBlock™ Blocking Buffer (Beyotime Biotechnology, #P0260) and incubated with anti-MUC2 (Abcam, #ab27269, dilutions 1:200), anti-CD64 (Abcam, #ab140779, dilutions 1:200) and anti-Occludin (Abcam, #ab216327, dilutions 1:200) antibodies overnight at 4 °C. Following washing with PBS, the sections were subjected to incubation with HRP-conjugated secondary antibodies at room temperature for 1.5 h. Color reaction was developed using the 3, 3-diaminobenzidine (DAB) Horseradish Peroxidase Color Development Kit (Beyotime Biotechnology, #P0202) for a period of 5 min, followed by counterstaining with hematoxylin. All stained slides were observed under a microscope and photographed after sealing with Polyvinylpyrrolidone Mounting Medium (Beyotime Biotechnology, #C0185). The HE staining procedure was conducted in accordance with standard protocol. Tissue sections were subjected to hematoxylin staining for 60 s at a temperature of 60 °C, followed by treatment with 1% ethanol hydrochloride for 3 s. Subsequently, eosin was applied to the sections, which were then rinsed with tap water. The slides were further processed by sequential treatment with ethanol and xylene for the appropriate duration, before being sealed with neutral gum. Representative images were captured using an optical microscope.

### Analysis of o-glycosylation

Endoscopic biopsies were obtained from non-inflammatory regions of four patients diagnosed with mild UC and four healthy individuals. Three biopsies (5–10 mg wet weight) were collected from each case to extract total protein, and protein concentrations were measured using spectrophotometry. Subsequently, the samples underwent testing via nanocapillary liquid chromatography-tandem mass spectrometry (nanoLC-MS/MS). In the investigation of O-linked glycopeptides within intestinal mucosal tissues, the pGlyco 2.0 search engine was utilized to identify matches of intact O-glycopeptides. Alkylation was designated as a static modification, while oxidation and acetylation were considered dynamic modifications. To obtain intact O-glycopeptide data, results were de-weighted based on modification, peptide sequence, and glycan composition. The intact O-glycopeptide was then identified by pGlyco 2.0. Additionally, Isotopic Envelope Fingerprinting (iEF) and secondary ion mass spectra were obtained to provide a visual representation of the structure.

### Fecal sample collection, nucleic acid extraction, and 16S rRNA gene sequencing

Fecal samples were collected and preserved using the Fecal Storage Kit, which contained 4 mL Stool Preservation Solution from Treatgut Biotechnology in Xiamen, China. Bacterial nucleic acids were extracted using the OMEGA Stool DNA Kit in accordance with the manufacturer's instructions. Primers were designed based on conserved regions of the 16S rDNA sequence for PCR analysis. The target fragments were recovered through agarose gel electrophoresis. Next, the purified library was quantified and sequenced (paired-end sequencing) on the Illumina platform 250PE following standard operations.

### Bioinformatics analysis

The acquisition of sequencing data and subsequent analysis of the gut microbiome were conducted utilizing Kraken2 software (version 2.0.7-beta). The taxonomic composition of each sample was evaluated, and a comparative analysis between groups was performed using linear discriminant analysis (LDA) effect size. Community structure and bacterial species distribution in different groups were visualized using GraPhlAn and python. Pathway annotation and analysis were conducted using HUMAnN2 software. Intergroup differences were further analyzed using analysis of variance (ANOVA).

### Bacterial growth assay

The bacteria were cultivated in LB medium devoid of antibiotics until the absorbance value reached approximately 0.6 at 600 nm. The bacterial solution was then distributed evenly into multiple portions and diluted with the same medium. Each group was subjected to triplicate wells. The mixed bacterial solution was incubated at 37 °C and 200 rpm/min with shaking. At hourly intervals, three samples were collected from each group and the bacterial absorbance values were measured to determine the concentration and quantity of bacteria. Growth curves were constructed for a minimum of 6 consecutive hours to evaluate the impact of diverse media or culture conditions on the proliferative capacity of the bacteria.

### Cell culture

Human HT29 epithelial colonic cells were procured from the cell bank of the Chinese Academy of Sciences located in Shanghai, China. The cells were maintained in DMEM supplemented with 10% fetal bovine serum and 1% penicillin/streptomycin at a temperature of 37 °C under high humidity conditions with 5% CO_2_.

### Bacterial adherence assay

HT29 cells in the logarithmic growth phase were cultured in 24-well dishes until reaching approximately 80% confluency, after which a bacterial adherence experiment was conducted. The bacterial precipitates (200 μL) were obtained via centrifugation, washed with PBS, and suspended in 400 μL of serum-free cell culture medium. The suspension was then incubated in an incubator shaker at 37 °C for 3 h. Subsequently, gradient dilution buffer was added to the bacterial solutions, followed by an additional 1 h of incubation. Next, the culture medium of the 24-well plates was discarded, and 400 μL of serum-free, antibiotic-free cell culture medium was added, followed by an incubation period of 1 h. Thereafter, 100 μL of bacterial solution with diverse dilution gradients was introduced into the aforementioned cell culture wells and permitted to incubate for a duration of 3 h. The supernatant was subsequently extracted, and the cells were rinsed with PBS before being gathered and resuspended in a sterile PBS solution. The resultant mixture was then transferred onto agar plates and incubated within a bacteriological incubator for a period of 12–16 h. The quantification of bacterial colonies grown on agar plates was based on the sizes of the colonies.

### Animal experiments

Male C57/BL6 mice, aged between 8 and 12 weeks, were procured from the Laboratory Animal Center of Nanjing University and housed in a specific pathogen-free (SPF) facility with a 12-h light/dark cycle. The mice were provided ad libitum access to food and water. The experimental procedures were conducted in compliance with institutional animal care guidelines and were approved by the Hospital Institutional Animal Care and Use Committee (IACUC) under approval number 2020JLHGKJDWLS-26. EPEC was suspended in 0.5 L of PBS. Mice were orally gavaged with 1.0 × 10^9^ colony-forming units (cfu) of EPEC or *E. coli* once daily for 7 consecutive days. In addition, the mice were intraperitoneally administered an O-glycan inhibitor (1 mg/kg). Body weights were recorded at 5-day intervals. After 15 days, the mice were euthanized under deep anesthesia and the colon was excised for length measurement and histological examination using hematoxylin and eosin (HE) staining.

### Western blotting

The tissue samples were pulverized using liquid nitrogen, lysed in pre-chilled RIPA buffer containing 1% protease inhibitor PMSF, and subsequently cooled on ice for 30 min. The lysates were then subjected to centrifugation at 12000 g and 4 °C for 12 min. The protein concentration was determined using the BCA method. For western blotting analysis, the samples were mixed with appropriate amounts of protein loading buffer, heated in a water bath at 95 °C for 10 min, and then cooled on ice. Standard SDS-PAGE gel electrophoresis was performed thereafter. The separated proteins were transferred to PVDF membranes. The membranes were blocked in 5% non-fat skimmed milk in TBST for 2 h and then incubated with anti-β-actin antibody (dilution 1:4000) overnight at 4 °C. After incubation with the secondary antibody, immunoblots were visualized by enhanced chemiluminescence and quantified using grayscale values. The protein expression of the housekeeping gene β-actin was utilized as an internal reference.

### qPCR analysis

The tissue samples were pulverized using liquid nitrogen. RNA extraction procedures were conducted on ice. The samples were lysed in 1 mL of TRIzol Reagent (Life Technologies) for 10 min, followed by chloroform extraction and isopropanol precipitation. The RNA precipitate was washed with 75% ethanol and dissolved in RNase-free water. The concentration and purity of RNA were assessed using a Nano-Drop Spectrophotometer. The TaqMan Reverse Transcription Kit was utilized to synthesize the cDNA, followed by qPCR using 2 × SYBR green. The 2^−ΔΔCt^ method was employed to analyze relative gene expression, which was normalized to GAPDH. The primer sequences for EPEC are provided below. lasR forward: 5ʹ-ATGGCCTTGGTTGACGGT-3ʹ, reverse: 5´-GCAAGATCAGAGAGTAATAAGACCC-3’; PvdA forward: 5’-GACCCATATGACACAGGCAACTGCAACCG-3ʹ, reverse: 5ʹ-CTATAAGCTTCAGCTGGCCAGGGCGTGC-3ʹ; pcrV forward: 5ʹ-TCTAGAATGGAAGTCAGAAACCTTAATGCC-3ʹ, reverse: 5ʹ-GTCGACGTAAATCTAGCGCGACTCTTACAGCGC-3ʹ.

### Statistical analysis

The data are presented as the mean ± standard deviation (SD) and statistical significance between two groups was assessed using unpaired Student’s t test through the utilization of SPSS software v.21 (IBM SPSS Statistics). Multigroup analysis was conducted through one-way ANOVA and LSD post-hoc test. Differences were deemed significant at *P* < 0.05. The significance levels for all data are indicated as follows: *P < 0.05; **P < 0.01; ***P < 0.001.

## Results

### Downregulation of Occludin in the intestinal epithelium of patients with mild UC

**Fig. 1:**
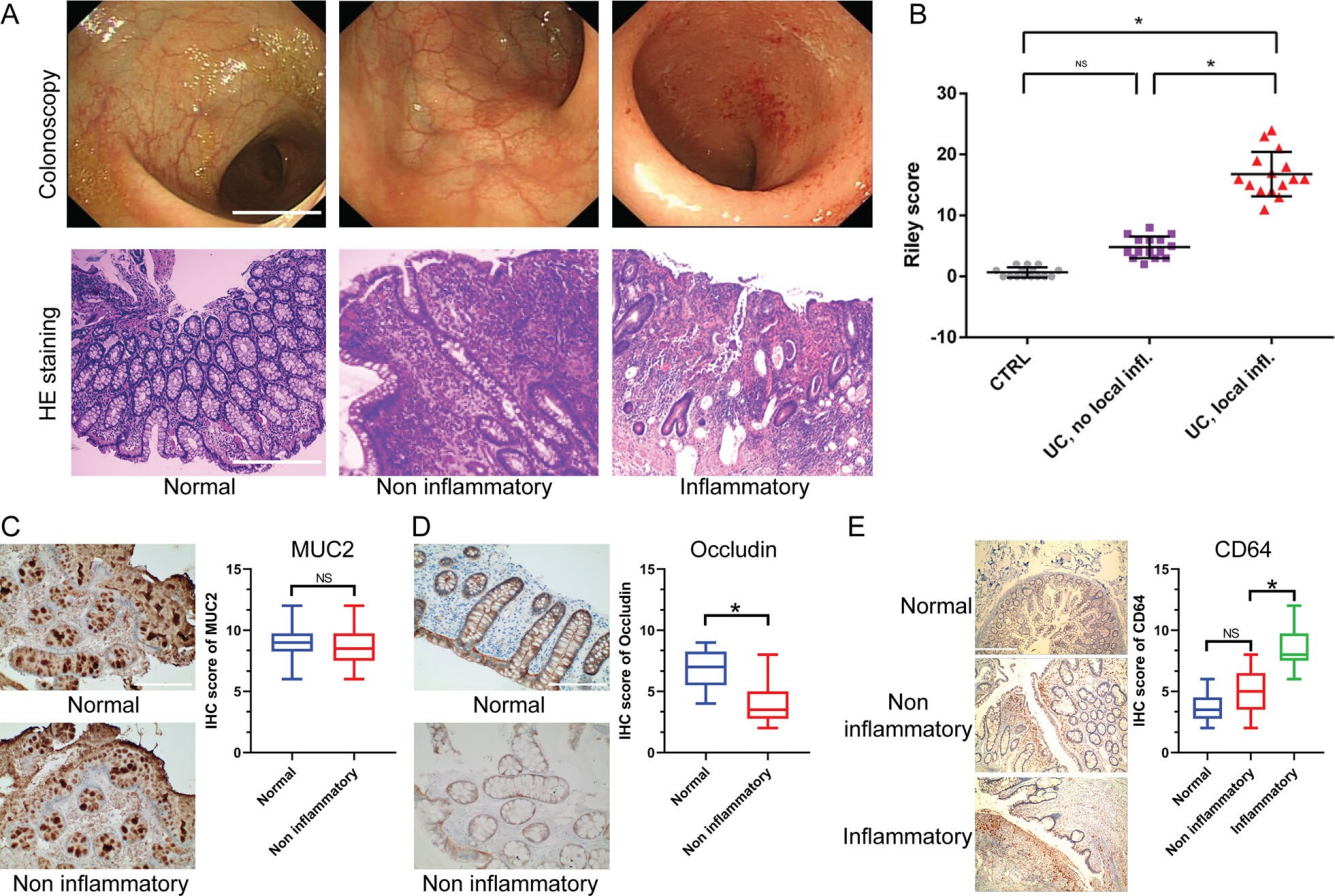
Inflammation and protein expressions within the intestinal epithelium of patients diagnosed with mild UC. **A** Endoscopic observations of colonic mucosa and HE staining (magnification, 20 ×) of tissue biopsies. **B** Statistical results of histological score. **C**-**E** Immunohistochemical staining displaying the protein expression of MUC2, Occludin, and CD64 within the intestinal epithelium. Statistical comparisons were determined by analysis of variance (ANOVA) among ≥ 3 groups or Student’s t test between two groups. NS, not significant; *P < 0.05

We conducted microscopic observations of both non-inflammatory and inflammatory tissues in patients with and without UC. The non-inflammatory sites of colonic mucosa in both non-UC patients and those with mild UC exhibited a smooth surface and visible vascular network. Conversely, at the inflammatory sites in patients with mild UC, we observed a superficial ulcer with congested mucosa (Fig. 1A, upper panel). The utilization of HE staining on biopsy-derived tissues revealed that the epithelium remained undamaged and that glandular branches were discernible in non-inflammatory regions of non-UC patients and those with mild UC. Nevertheless, inflammatory cell infiltration was evident in non-inflammatory regions of mild UC patients. It is noteworthy that patients with mild UC displayed epithelial destruction, glandular branching, crypt abscesses, and substantial inflammatory cell infiltration at inflammatory sites (Fig. 1A, lower panel). The histologic score indicated a greater degree of inflammation in UC-afflicted sites compared to non-inflammatory sites and non-UC patients. Notably, no significant difference was observed between non-inflammatory sites and controls (Fig. 1B). Immunohistochemical analysis revealed comparable MUC2 protein expression levels in non-inflammatory sites of non-UC patients and mild UC patients. However, Occludin protein expression was significantly lower in non-inflammatory sites relative to controls (Fig. 1C and D). Besides, a significant quantity of CD64^+^ monocyte/macrophage infiltrates were observed in the intestinal epithelium of patients with mild UC, both at inflammatory and non-inflammatory sites, in comparison to healthy controls (Fig. 1E).

### o-glycosylation of MUC2 in the intestinal epithelium of patients with mild UC

**Fig. 2:**
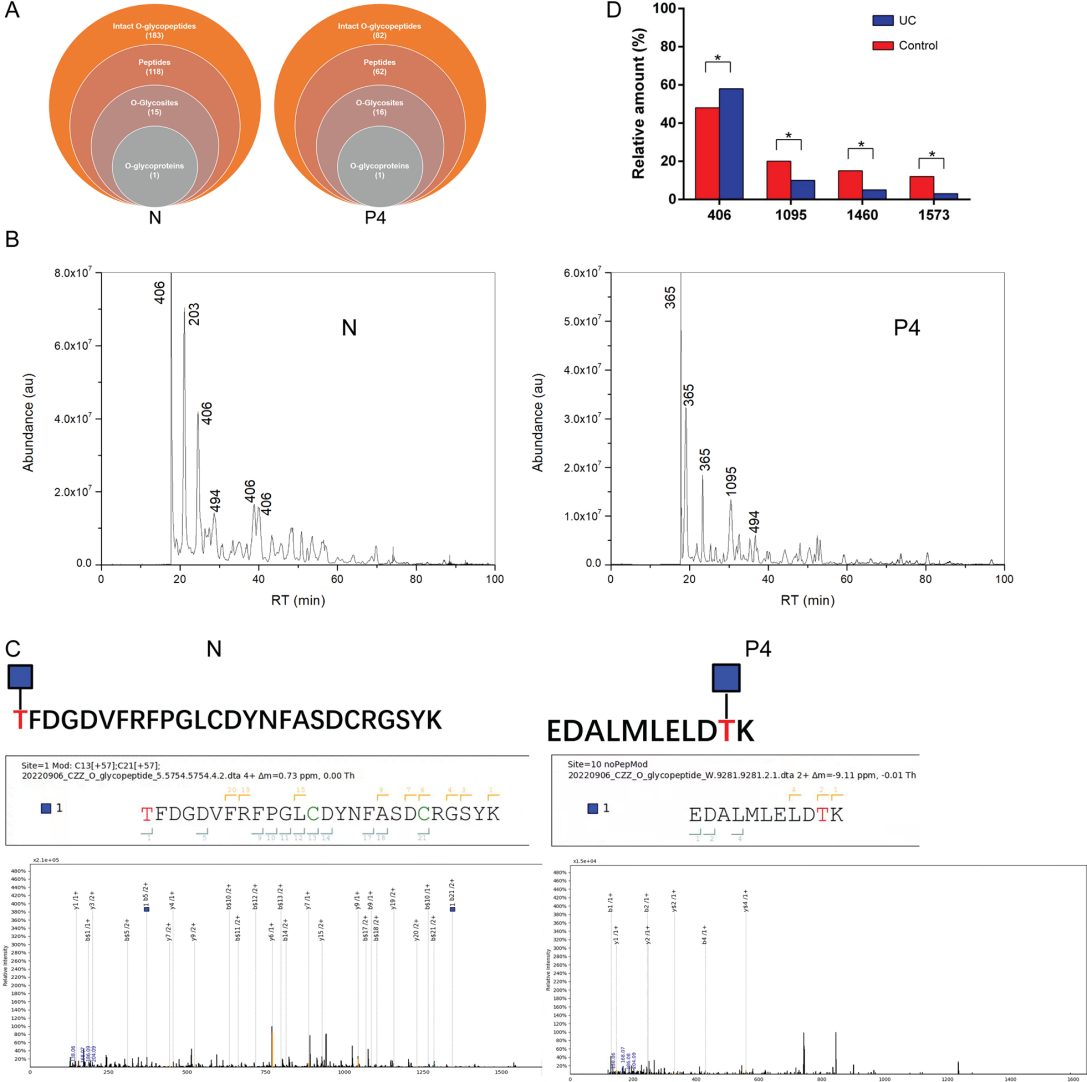
Intact O-glycopeptides identification by pGlyco 2.0. **A** Venn diagram showing intact O-glycopeptides in tissues samples. **B** The abundance of O-glycans in tissue samples. **C** The binding of intact O-glycopeptides to MUC2 protein in tissue samples. **A**, **B**, and **C**: left, normal control; right, the fourth patient with UC. **D** Relative abundance of several key glycans of MUC2 O-glycan in control and UC samples. N, tissues from healthy subjects. P4, tissue obtained from the fourth patient with UC. Differences between two groups were assessed using Student’s t-test. *P < 0.05

The absence of a notable variance in MUC2 protein expression at non-inflammatory sites between non-UC patients and mild UC patients, coupled with a reduction in Occludin protein levels, implies that the colonic epithelial structure remains largely unaltered in mild UC patients. However, it is plausible that the permeability of the structure may have been affected. To delve deeper into the underlying mechanisms, we conducted an investigation into the glycosylation level of the colonic epithelium in both groups, utilizing nanoLC-MS/MS. The analysis of intact O-glycopeptides was conducted using pGlyco 2.0 (Fig. 2A; Additional file 1: Figure S1), which facilitated the detection of various O-glycan abundances in tissue samples (Fig. 2B; Additional file 1: Figure S1). The control group exhibited a distinctive glycan structure, characterized by a relatively lower proportion of shorter glycan chains and a higher proportion of complex glycans, in comparison to the UC group (Fig. 2C; Additional file 1: Figure S1). The semiquantitative evaluation of individual oligosaccharides of colonic MUC2 in the two groups was conducted based on their mass-to-charge ratio (m/z) value. The relative quantities of four key glycans, identified by their molecular mass, were determined by the binding strength of single- and double-charged ions. The glycopeptides in the UC group exhibited distinct structural patterns, characterized by a reduction in longer glycan chains, such as 1095 (10%), 1460 (5%), and 1573 (4%) compared to the control group. However, the UC group showed a 13% higher relative amount of TF antigen (glycans 406) than the control group (Fig. 2D).

### Significant alterations in intestinal microbiota composition and higher abundance of *Escherichia coli* in patients with mild UC

**Fig. 3:**
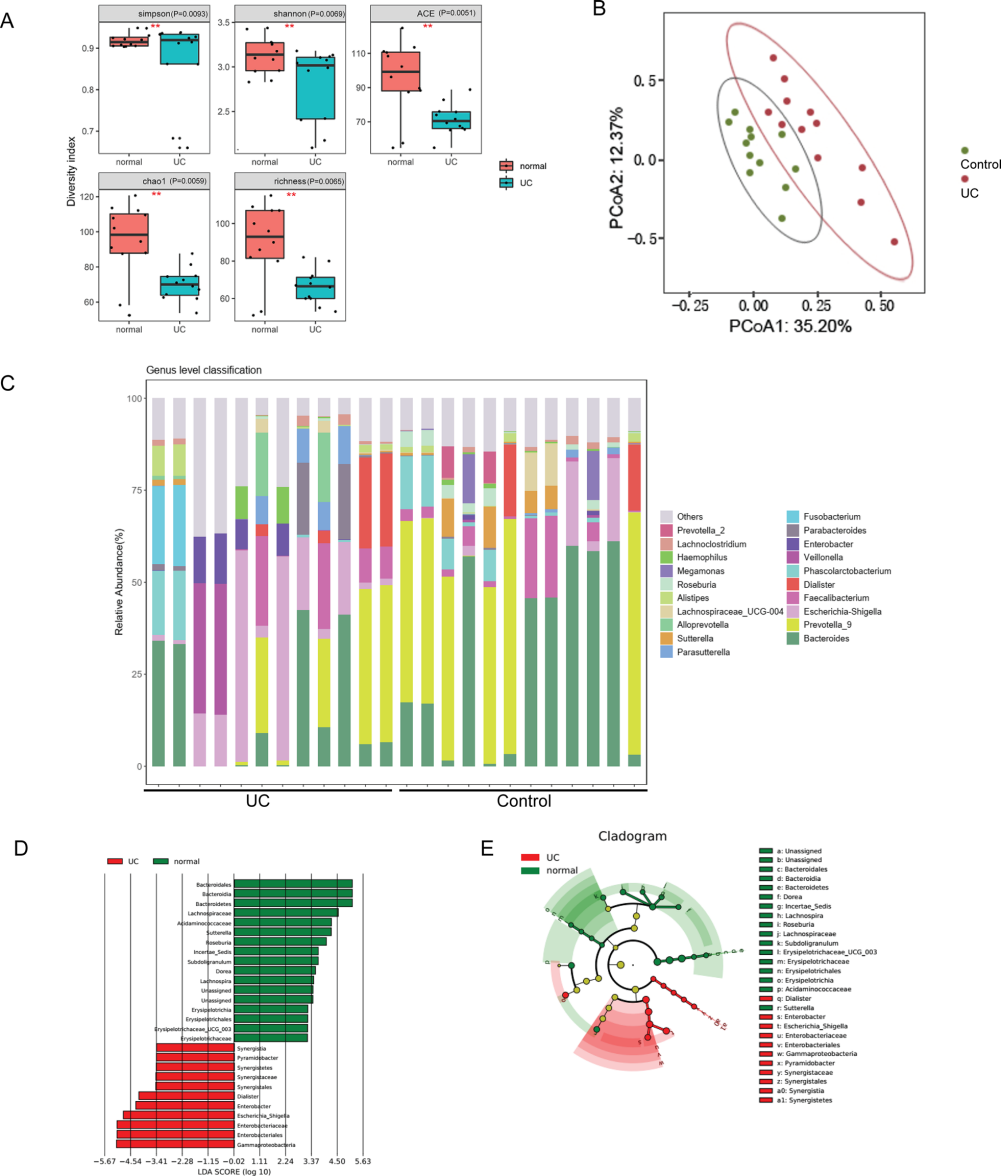
The composition of intestinal microbiota in the UC and control groups. **A** The α-diversity, richness (Chao1 and ACE indexes) and microbial-diversity (Shannon and Simpson indexes) of gut microbiota in the UC and control groups. **B** Microbial β-diversity in the UC and control groups based on principal coordinates analysis (PCoA). **C** The abundance of microbial taxa at genus level. **D** Histogram of LDA scores between the UC and control groups. **E** The differences in microbial taxa between the UC and control groups based on LEfSe analysis

Prior research has indicated a correlation between O-glycan modifications in the colonic mucosa and the distribution of intestinal microbiota. To investigate the distinctions in the distribution of intestinal microbiota between individuals with mild UC and healthy subjects, an analysis of the gut bacterial community was conducted through 16S rRNA gene sequencing. The results revealed that the α-diversity indices of the gut microbiome, including Simpson (P = 0.0093), Shannon (P = 0.0069), Chao1 (P = 0.0059), ACE (P = 0.0051), and richness (P = 0.0065) indices, exhibited a significantly lower diversity in UC patients than in healthy individuals (Fig. 3A). Additionally, the β-diversity, as determined by Principle Component Analysis (PCoA), was also lower in UC patients (Fig. 3B). The composition of the gut bacterial community was summarized at various taxonomic levels, including genus, phylum, class, order, and family (Fig. 3C; Additional file 2: Figure S2). To identify significant biomarkers, LDA was conducted, revealing a significant difference in the abundance of *E. coli* between the two groups (Fig. 3D). Furthermore, Linear Discriminate Analysis effect size (LEfSe) detected 28 taxa with differential abundance (LDA score > 2.0) between patients with UC and healthy controls (Fig. 3E).

### The supernatant of intestinal epithelial cells affects *Escherichia coli* propagation

**Fig. 4:**
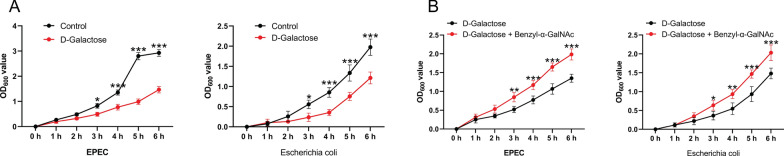
The growth of *Escherichia coli* is influenced by the supernatant of intestinal epithelial cells. **A** The proliferative capacity of both pathogenic and commensal *E. coli* is affected by the supernatant of D-galactose-induced HT29 cells. **B** The impact of D-galactose-induced HT29 cells and/or the O-glycan synthesis inhibitor benzyl-α-GalNAc-treated cell supernatant on the proliferative capacity of pathogenic and commensal *E. coli*. Differences between two groups were assessed using Student’s t-test. *P < 0.05; **P < 0.01; ***P < 0.001

To explore the interplay between O-glycans, *E. coli*, and the intestinal epithelium, we employed established techniques that utilize D-galactose to prompt HT29 cells to differentiate into mucus-secreting, highly specialized epithelial cells. The resulting supernatant was subsequently combined with *E. coli* LB medium and utilized to cultivate pathogenic *E. coli* or *E. coli* obtained from healthy human feces, with the aim of assessing the impact on bacterial proliferative capacity. Our findings indicated that the proliferative capacity of both pathogenic and commensal *E. coli* was significantly suppressed by the D-galactose-induced cell supernatant (Fig. 4A). Nevertheless, the inhibitory effects on *E. coli* proliferation were reversed upon treatment with the O-glycan synthesis inhibitor, benzyl-α-GalNAc, in comparison to D-galactose treatment alone (Fig. 4B).

### The supernatant of intestinal epithelial cells affects the adhesion of *Escherichia coli*

**Fig. 5:**
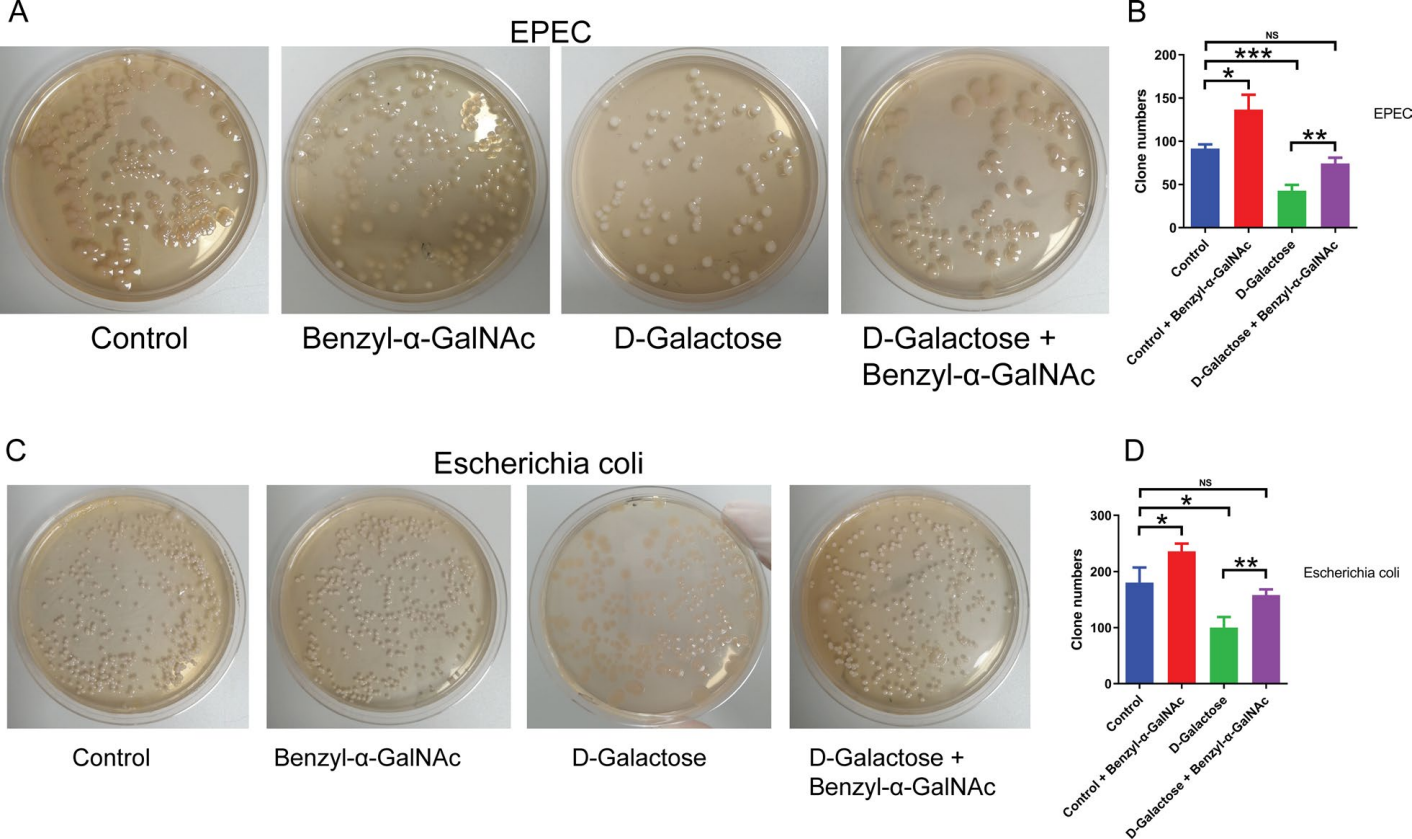
The adhesive capacity of *Escherichia coli* is influenced by the supernatant of cell culture. (**A**, **B**) Effect of cell culture supernatant on the adhesive capacity of EPEC *E. coli*. (**C**, **D**) Effect of cell culture supernatant on the adhesive capacity of commensal *E. coli*. HT29 cells were treated with benzyl-α-GalNAc and/or D galactose for 48 h. EPEC, enteropathogenic *Escherichia coli*. Differences among the multiple groups were analyzed by one-way ANOVA. NS, not significant; *P < 0.05; **P < 0.01; ***P < 0.001

The adhesion capacity of bacteria to epithelial cells is a crucial determinant of their pathogenicity. This study aimed to investigate the adhesion capabilities of enteropathogenic *E. coli* and commensal *E. coli*. To this end, we collected cell culture supernatant from HT29 cells that were subjected to various treatments, including an O-glycosylation inhibitor (benzyl-α-GalNAC), highly differentiated HT29 cells induced with D-galactose, HT29 cells co-treated with both benzyl-α-GalNAc and D-galactose, and untreated cells (control). Subsequently, the two *E. coli* strains were subjected to the treatments mentioned above. Our findings indicated a significant increase in the adhesion capacity of both *E. coli* strains to cells treated with benzyl-α-GalNAc cell supernatant, while a decrease in adhesion capacity was observed in both *E. coli* strains to D-galactose-induced highly differentiated HT29 cell supernatant compared to HT29 cell supernatant (Fig. 5A, B). Furthermore, the cell supernatant obtained from D-galactose and benzyl-α-GalNAc-cotreated HT29 cells was able to reverse the adhesion ability of both *E. coli* strains in comparison to that induced by D-galactose alone (Fig. 5C, D).

### Co-treatment with *Escherichia coli* and the O-glycan inhibitor benzyl-α-GalNAc promotes the development of colitis in mice

**Fig. 6:**
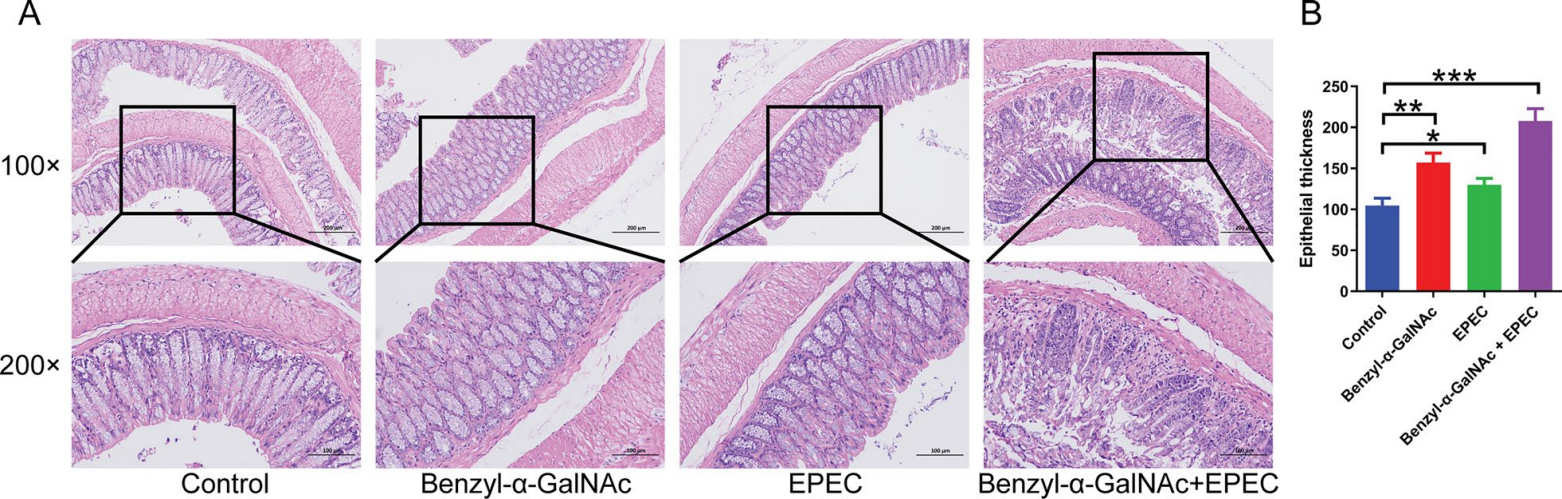
Co-treatment with *Escherichia coli* and O-glycan inhibitor benzyl-α-GalNAc promotes colitis in mice. **A** The effects of benzyl-α-GalNAc and/or EPEC *E. coli* on colonic thickening in mice. **B** Statistical results. The mice were subjected to intraperitoneal injections of O-glycan inhibitor benzyl-α-GalNAc (1 mg/kg) and/or EPEC *E. coli* by gavage (1 × 109 cfu/mL in sterile PBS) once daily for 7 consecutive days. IP, intraperitoneal injections; cfu, colony forming units. Differences among the multiple groups were analyzed by one-way ANOVA. *P < 0.05; **P < 0.01; ***P < 0.001

To further validate the interplay among O-glycans, *E. coli*, and the intestinal epithelium, we administered the O-glycosylation inhibitor benzyl-α-GalNAc via intraperitoneal (i.p.) injection and/or EPEC *E. coli* via gavage to the murine subjects. benzyl-α-GalNAc treatment alone elicited a significant thickening of the colon in mice compared to the control. Similarly, the administration of EPEC *E. coli* via gavage resulted in an increase in colonic thickening in mice; however, the extent of alteration was less pronounced than that observed in the benzyl-α-GalNAc-treated cohort. In addition, the co-administration of EPEC *E. coli* and benzyl-α-GalNAc in mice resulted in a noteworthy augmentation of colonic thickness, concomitant with colonic epithelial impairment, disrupted glandular architectures, crypt abscesses, and extensive infiltration of inflammatory cells (Fig. 6).

### *Escherichia coli* downregulates glycosyltransferase C1GALT1 expression in HT29 cells by modulating the NF-κB pathway

**Fig. 7:**
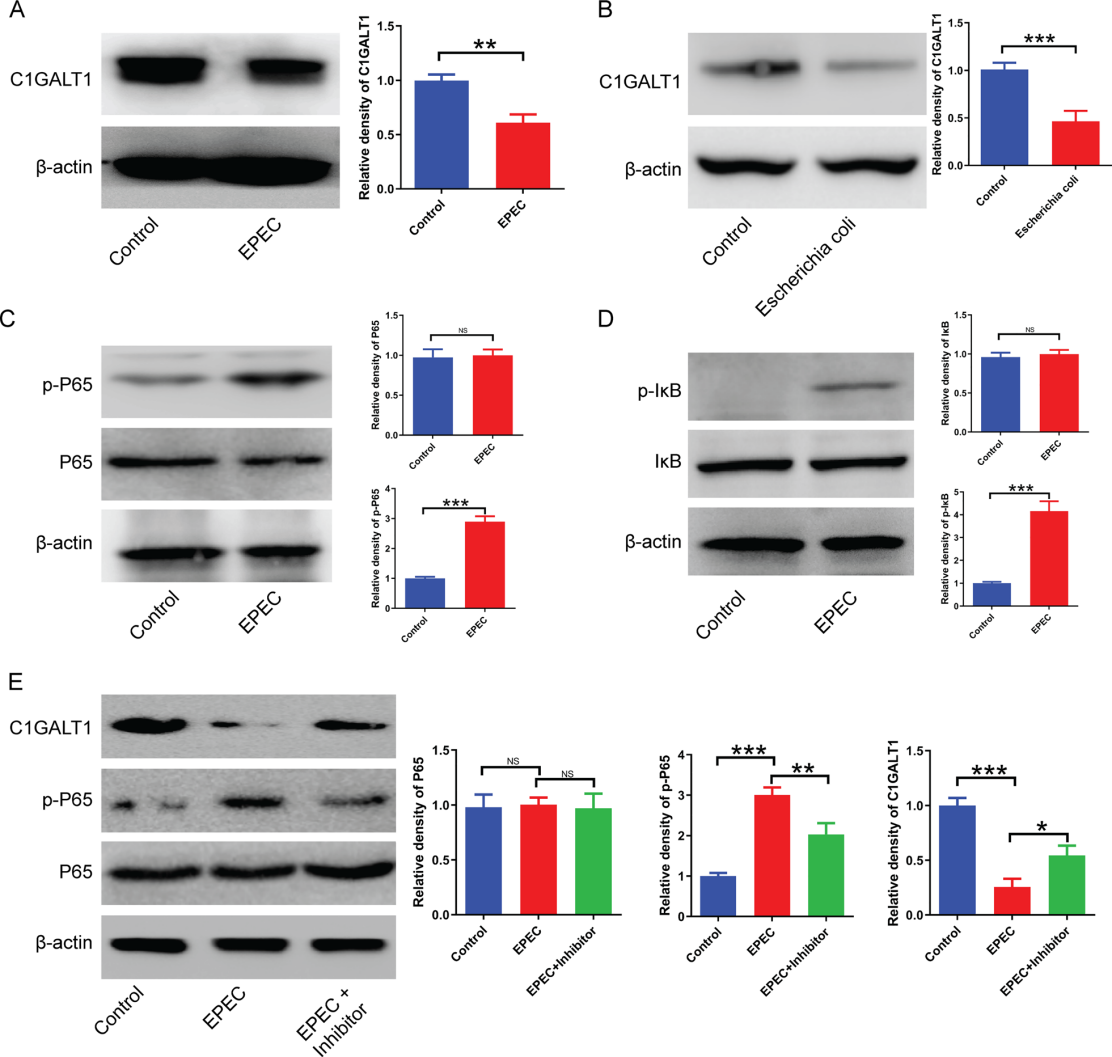
The expression of glycosyltransferase C1GALT1 is down-regulated by *Escherichia coli* through the NF-κB pathway. **A**, **B** The impact of both enteropathogenic and commensal *E. coli* on C1GALT1 expression in HT29 cells. **C**, **D** The effects of EPEC on the phosphorylation levels of cellular P65 and IκB. **E** The levels of P65, p-P65, and C1GALT1 after co-treatment of cells with EPEC and/or NF-κB inhibitor BAY 11–7082. EPEC, enteropathogenic *Escherichia coli*. Statistical comparisons were determined by analysis of variance (ANOVA) among ≥ 3 groups or Student’s t test between two groups. NS, not significant; *P < 0.05; **P < 0.01; ***P < 0.001

Our study revealed a correlation between O-glycans, *E. coli*, and the intestinal epithelium. Nonetheless, the precise mechanisms underlying the influence of *E. coli* on O-glycan biosynthesis in the intestinal epithelium, which ultimately leads to the development of UC, necessitate further investigation. In light of this, we performed a co-culture experiment involving *E. coli* and HT29 cells to evaluate the effects of bacterial activity on cellular signaling molecules and protein expression. The data presented in this study indicate that both EPEC *E. coli* and commensal *E. coli* significantly decreased the expression of glycosyltransferase C1GALT1, a crucial enzyme for O-glycan synthesis, when compared to untreated cells (Fig. 7A, B). Additionally, *E. coli* induced the phosphorylation of P65 and IκB, two key molecules in the NF-κB pathway, without significantly affecting the total levels of P65 and IκB (Fig. 7C, D). Furthermore, co-treatment of the cells with the NF-κB inhibitor BAY 11–7082 and EPEC significantly reduced the levels of p-P65 and restored C1GALT1 expression compared to cells treated with EPEC alone (Fig. 7E).

### The NF-κB pathway mediates the effects of *Escherichia coli* and o-glycans on UC development in mice

**Fig. 8:**
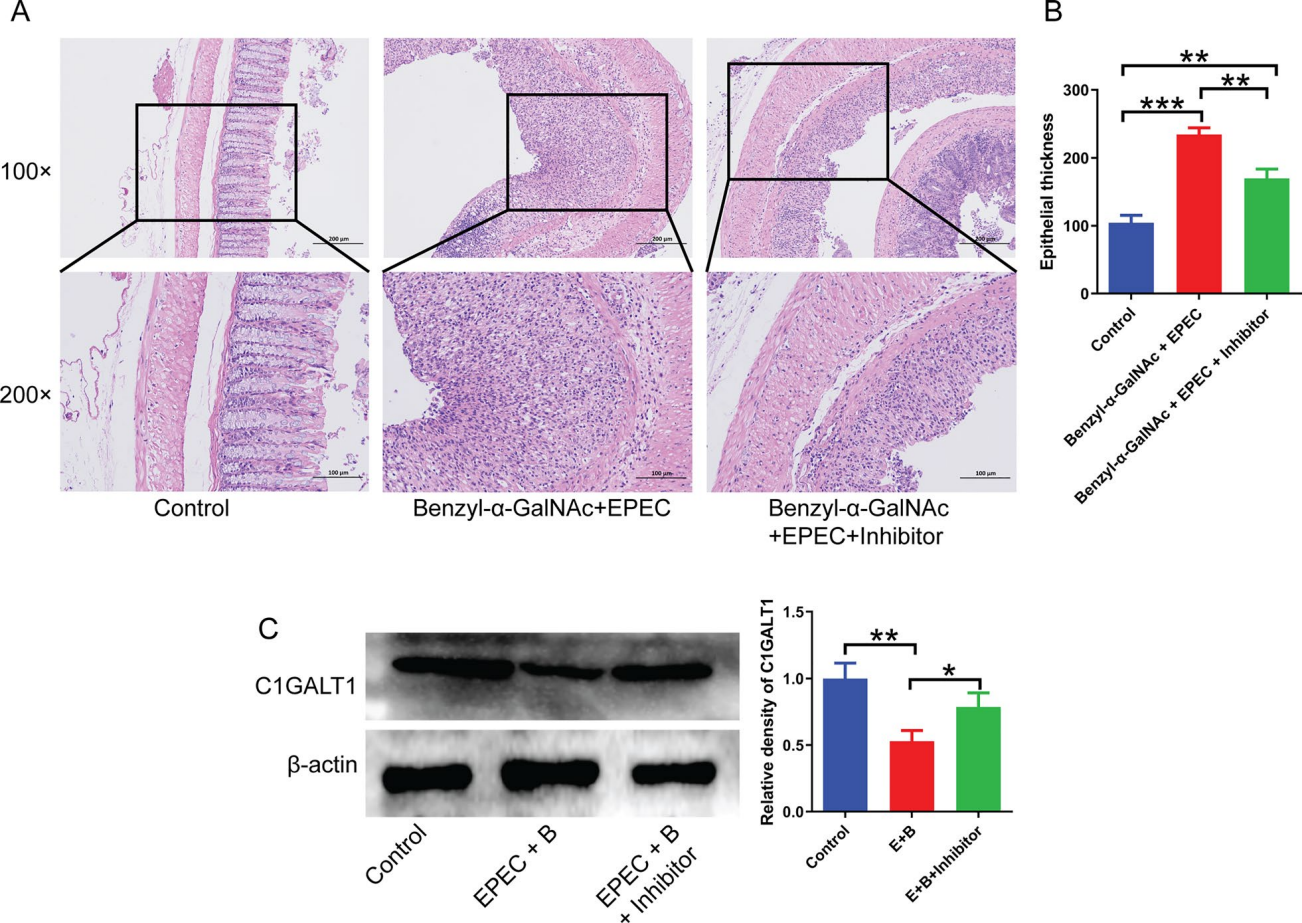
The NF-κB pathway serves as a mediator for the impact of *E. coli* and O-glycan on ulcerative colitis in mice. **A** and **B** The effects of various treatments on colonic thickening in mice were demonstrated through HE staining. **C** The protein expression of C1GALT1 in the colon of mice. Differences among the multiple groups were analyzed by one-way ANOVA. *P < 0.05; **P < 0.01; ***P < 0.001

To validate the involvement of the NF-κB pathway in the pathogenesis of *E. coli*-induced ulcerative colitis, we conducted additional animal experiments. Our findings were in agreement with the aforementioned results, as mice that received co-treatment with EPEC and benzyl-α-GalNAc exhibited augmented colonic thickening and inflammatory infiltration relative to the control group. However, co-administration of the NF-κB inhibitor BAY 11-7082 resulted in a significant reduction in colonic thickening and inflammatory infiltration compared to mice that were solely exposed to EPEC (Fig. 8A, B). Besides, the concurrent administration of EPEC and benzyl-α-GalNAc resulted in a notable decrease in the expression of C1GALT1, whereas the utilization of the NF-κB inhibitor BAY 11-7082 effectively restored the protein expression of C1GALT1 (Fig. 8C).

MUC2 constitutes a pivotal constituent within the mucous layer. Nevertheless, there exist other indispensable proteins within the mucus barrier, such as TFF3 and FCGBP. The TFF protein family, primarily secreted by gastrointestinal cells, assumes a fundamental role in upholding the integrity of the intestinal barrier. TFF3, as a prominent constituent, substantially contributes to the rectification of damage incurred by the intestinal epithelium and the regulation of inflammatory processes. Additionally, FCGBP, closely associated with TFF3, reinforces the defenses of the mucus barrier, particularly in the context of microbial infections [26]. In the present investigation, a significant reduction in the levels of TFF3 and FCGBP expression was noted in the intestinal tissues of mice subjected to EPEC treatment, in comparison to the untreated mice (Additional file 3: Figure S3). Additionally, administration of the NF-κB inhibitor BAY 11-7082 did not yield a statistically noteworthy effect on mice exposed to EPEC in comparison to those treated solely with EPEC (Additional file 4: Figure S4). These findings suggest that the downregulation of TFF3 and FCGBP triggered by EPEC operates via a mechanism that is distinct and unrelated to the NF-κB signaling pathway.

## Discussion

Ulcerative colitis is a chronic inflammatory disease that primarily impacts the colon and rectum [2, 27]. While genetic factors, host immune system disorders, dysbiosis of the intestinal microbiota, and environmental factors have been associated with UC pathogenesis in recent research [3–5], the molecular mechanisms underlying UC development remain incompletely understood. Consequently, additional investigation into potential therapeutic strategies may enhance patient prognosis.

The intestinal mucus barrier serves as the primary defense mechanism against environmental, physiological, and immune stimuli, and its impairment is a significant manifestation of UC [17, 20]. The colonic mucus layer primarily comprises mucin, while proteoglycan components play a crucial role in preserving the mucus barrier, which undergoes significant alterations during the development of UC [14, 15, 17]. In this study, we mainly observed MUC2 protein expression in colonic tissues of patients with mild UC, and no significant changes were observed. MUC2 serves as a marker for intestinal epithelial permeability [28, 29], and the less severe manifestation of lesions in patients with mild UC may be attributed to the absence of significant structural changes. However, our findings indicated a significant reduction in the expression of the tight junction protein Occludin, which is indicative of mucosal damage and compromised gut epithelial barrier integrity [30–32]. It was subsequently confirmed that protein glycosylation was markedly diminished in individuals with UC, indicating a potential association between UC, protein glycosylation, and mucosal permeability. Taken together, these findings suggest that mild UC patients exhibit heightened intestinal permeability despite preservation of the intestinal epithelial barrier.

The colonic mucus layer serves as the primary dwelling place for commensal bacteria. The composition of gut microbiota is under the regulation of the intestinal mucus layer, and the host mucus has the potential to shape the intestinal microbiota [33–35]. The gut microbiota of individuals with IBD differs from that of healthy individuals and may play a crucial role in the pathogenesis of IBD [33, 36]. In line with this, our study also revealed significant variations in the intestinal microbiota between healthy controls and patients with mild, severe, and severe UC. Next, we directed our attention toward *E. coli*, an opportunistic pathogen, amidst the varied abundance of microbial taxa. *E. coli* typically operates as a commensal bacterium in regular circumstances, but its role shifts to that of a harmful pathogen when the host's immune system weakens, leading to the onset of disease [37–39]. The potential influence of O-glycans in promoting heightened virulence and invasiveness of *E. coli* has been suggested [14, 40]. Our data indicated that the treatment of HT29 cells with an O-glycan inhibitor enhanced the proliferative capacity and adhesion of *E. coli*. While the specific molecular mechanism behind these effects remains unclear, our findings suggested that post-translational glycosylation modifications influenced the phenotypes of commensal gut microbiota, thereby impacting disease progression, including UC, through host interactions. Prior research has demonstrated that the elimination of glycosyltransferases resulted in a marked decrease in O-glycan manifestation, which subsequently led to the onset of intestinal inflammation and colorectal cancer in mice [23–25]. Our animal experimentation similarly revealed that the administration of an O-glycan inhibitor induced epithelial thickening, and concurrent treatment with *E. coli* intensified colonic inflammation in mice. As previously stated, the pathogenesis of UC is triggered by impairment of barrier integrity in intestinal mucosa and gut microbiota dysbiosis [33, 34]. Our findings support this notion, as co-culturing *E. coli* with epithelial cells resulted in significant activation of the NF-κB pathway and marked down-regulation of glycosyltransferase C1GALT1 protein expression in the epithelium, leading to a decrease in O-glycan expression. In contrast, the administration of an inhibitor targeting the NF-κB pathway resulted in the reversal of the aforementioned alterations, which were partially replicated in animal models. Prior investigations have demonstrated a strong correlation between the NF-κB pathway and IBD [41–43], a finding that is supported by our current findings.

## Conclusions

In summary, our findings provide confirmation that a decrease in E. coli levels within the gastrointestinal tract could potentially be linked to O-glycosylation. It is widely acknowledged that patients with UC exhibit reduced O-glycosylation, yet the impact of this phenomenon on the composition and prevalence of intestinal flora, specifically E. coli, is a relatively recent concept. Moreover, the altered abundance of E. coli in the intestines, subsequently influencing the activity of the NFKB signaling pathway in the mucosal lining of the host, offers a fresh perspective on the microecology of the intestines and the progression of UC.

### Supplementary Information

**Additional file 1: Figure S1.** Identification of intact O-glycopeptides in intestinal tissues obtained from three additional patients with UC. (A) Venn diagram illustrating the presence of intact O-glycopeptides in tissues samples of patients with UC. (B) The abundance of O-glycans in tissue samples of patients with UC. (C) The binding of intact O-glycopeptides to MUC2 protein in tissue samples of patients with UC. P represents tissues obtained from patients with UC.

**Additional file 2: Figure S2.** The relative abundance of fecal microbiota identified at the phylum, class, order, and family levels.

**Additional file 3: Figure S3. **The protein levels of TFF3 and FCGBP in intestinal tissues of EPEC-treated mice. Differences between two groups were assessed using Student’s t-test. **P< 0.01; ***P< 0.001.

**Additional file 4: Figure S4.** The protein levels of TFF3 and FCGBP in intestinal tissues of EPEC E. coli and benzyl-α-GalNAc cotreated mice. Differences among the multiple groups were analyzed by one-way ANOVA. *P< 0.05; **P< 0.01; ***P< 0.001.

## Data Availability

The datasets used and/or analyzed during the current study are available from the corresponding author on reasonable request.

## Abbreviations

UC: Ulcerative colitis
CD: Crohn disease

## References

[1] Kaplan GG (2015). The global burden of IBD: from 2015 to 2025. Nat Rev Gastroenterol Hepatol.

[2] Du L, Ha C (2020). Epidemiology and pathogenesis of ulcerative colitis. Gastroenterol Clin North Am.

[3] Segal JP, LeBlanc JF, Hart AL (2021). Ulcerative colitis: an update. Clin Med.

[4] Wei SC, Sollano J, Hui YT, Yu W, Santos Estrella PV, Llamado LJQ (2021). Epidemiology, burden of disease, and unmet needs in the treatment of ulcerative colitis in Asia. Expert Rev Gastroenterol Hepatol.

[5] Piovani D, Danese S, Peyrin-Biroulet L, Nikolopoulos GK, Lytras T, Bonovas S (2019). Environmental risk factors for inflammatory bowel diseases: an umbrella review of meta-analyses. Gastroenterology.

[6] Gearry RB (2016). IBD and environment: are there differences between East and West. Digest Dis.

[7] Misselwitz B, Juillerat P, Sulz MC, Siegmund B, Brand S (2020). Emerging treatment options in inflammatory bowel disease: janus kinases, stem cells, and more. Digestion.

[8] Burri E, Maillard MH, Schoepfer AM, Seibold F, Van Assche G, Rivière P (2020). Treatment algorithm for mild and moderate-to-severe ulcerative colitis: an update. Digestion.

[9] Singh S, Allegretti JR, Siddique SM, Terdiman JP (2020). AGA technical review on the management of moderate to severe ulcerative colitis. Gastroenterology.

[10] Fornaro R, Caratto M, Barbruni G, Fornaro F, Salerno A, Giovinazzo D (2015). Surgical and medical treatment in patients with acute severe ulcerative colitis. J Dig Dis.

[11] Liu S, Eisenstein S (2021). State-of-the-art surgery for ulcerative colitis. Langenbecks Arch Surg.

[12] Jangi S, Holmer AK, Dulai PS, Boland BS, Collins AE, Pham L (2021). Risk of relapse in patients with ulcerative colitis with persistent endoscopic healing: a durable treatment endpoint. J Crohns Colitis.

[13] Yilmaz B, Juillerat P, Øyås O, Ramon C, Bravo FD, Franc Y (2019). Microbial network disturbances in relapsing refractory Crohn’s disease. Nat Med.

[14] Yao D, Dai W, Dong M, Dai C, Wu S (2021). MUC2 and related bacterial factors: therapeutic targets for ulcerative colitis. EBioMedicine.

[15] McGuckin MA, Hasnain SZ (2014). There is a 'uc' in mucus, but is there mucus in UC?. Gut.

[16] Fang J, Wang H, Zhou Y, Zhang H, Zhou H, Zhang X (2021). Slimy partners: the mucus barrier and gut microbiome in ulcerative colitis. Exp Mol Med.

[17] van der Post S, Jabbar KS, Birchenough G, Arike L, Akhtar N, Sjovall H (2019). Structural weakening of the colonic mucus barrier is an early event in ulcerative colitis pathogenesis. Gut.

[18] Guo XY, Liu XJ, Hao JY (2020). Gut microbiota in ulcerative colitis: insights on pathogenesis and treatment. J Dig Dis.

[19] Shen ZH, Zhu CX, Quan YS, Yang ZY, Wu S, Luo WW (2018). Relationship between intestinal microbiota and ulcerative colitis: mechanisms and clinical application of probiotics and fecal microbiota transplantation. World J Gastroenterol.

[20] Niv Y (2016). Mucin gene expression in the intestine of ulcerative colitis patients: a systematic review and meta-analysis. Eur J Gastroenterol Hepatol.

[21] Werlang CA, Chen WG, Aoki K, Wheeler KM, Tymm C, Mileti CJ (2021). Mucin O-glycans suppress quorum-sensing pathways and genetic transformation in Streptococcus mutans. Nat Microbiol.

[22] Yamada T, Hino S, Iijima H, Genda T, Aoki R, Nagata R (2019). Mucin O-glycans facilitate symbiosynthesis to maintain gut immune homeostasis. E Bio Med.

[23] Fu J, Wei B, Wen T, Johansson ME, Liu X, Bradford E (2011). Loss of intestinal core 1-derived O-glycans causes spontaneous colitis in mice. J Clin Investig.

[24] Bergstrom K, Liu X, Zhao Y, Gao N, Wu Q, Song K (2016). Defective intestinal mucin-type O-glycosylation causes spontaneous colitis-associated cancer in mice. Gastroenterology.

[25] An G, Wei B, Xia B, McDaniel JM, Ju T, Cummings RD (2007). Increased susceptibility to colitis and colorectal tumors in mice lacking core 3-derived O-glycans. J Exp Med.

[26] Song C, Chai Z, Chen S, Zhang H, Zhang X, Zhou Y (2023). Intestinal mucus components and secretion mechanisms: what we do and do not know. Exp Mol Med.

[27] Suzuki Y (2017). The diagnostic criteria and differential diagnosis of IBD. Nihon rinsho Jpn J Clin Med.

[28] Yamashita MSA, Melo EO (2018). Mucin 2 (MUC2) promoter characterization: an overview. Cell Tissue Res.

[29] Johansson ME, Larsson JM, Hansson GC (2011). The two mucus layers of colon are organized by the MUC2 mucin, whereas the outer layer is a legislator of host-microbial interactions. Proc Natl Acad Sci USA.

[30] Saito AC, Higashi T, Fukazawa Y, Otani T, Tauchi M, Higashi AY (2021). Occludin and tricellulin facilitate formation of anastomosing tight-junction strand network to improve barrier function. Mol Biol Cell.

[31] Richter JF, Hildner M, Schmauder R, Turner JR, Schumann M, Reiche J (2019). Occludin knockdown is not sufficient to induce transepithelial macromolecule passage. Tissue barriers.

[32] Rawat M, Nighot M, Al-Sadi R, Gupta Y, Viszwapriya D, Yochum G (2020). IL1B increases intestinal tight junction permeability by up-regulation of MIR200C-3p, which degrades occludin mRNA. Gastroenterology.

[33] Kudelka MR, Stowell SR, Cummings RD, Neish AS (2020). Intestinal epithelial glycosylation in homeostasis and gut microbiota interactions in IBD. Nat Rev Gastroenterol Hepatol.

[34] Desai MS, Seekatz AM, Koropatkin NM, Kamada N, Hickey CA, Wolter M (2016). A dietary fiber-deprived gut microbiota degrades the colonic mucus barrier and enhances pathogen susceptibility. Cell.

[35] Chen Z, Luo J, Li J, Kim G, Chen ES, Xiao S (2021). Foxo1 controls gut homeostasis and commensalism by regulating mucus secretion. J Exp Med.

[36] Larabi A, Barnich N, Nguyen HTT (2020). New insights into the interplay between autophagy, gut microbiota and inflammatory responses in IBD. Autophagy.

[37] Palmela C, Chevarin C, Xu Z, Torres J, Sevrin G, Hirten R (2018). Adherent-invasive *Escherichia coli* in inflammatory bowel disease. Gut.

[38] Mirsepasi-Lauridsen HC, Vallance BA, Krogfelt KA, Petersen AM (2019). *Escherichia coli* pathobionts associated with inflammatory bowel disease. Clin Microbiol Rev.

[39] Perna A, Hay E, Contieri M, De Luca A, Guerra G, Lucariello A (2020). Adherent-invasive *Escherichia coli* (AIEC): Cause or consequence of inflammation, dysbiosis, and rupture of cellular joints in patients with IBD?. J Cell Physiol.

[40] Hansson GC (2020). Mucins and the microbiome. Annu Rev Biochem.

[41] Lin JC, Wu JQ, Wang F, Tang FY, Sun J, Xu B (2019). QingBai decoction regulates intestinal permeability of dextran sulphate sodium-induced colitis through the modulation of notch and NF-κB signalling. Cell Prolif.

[42] Chen X, Liu G, Yuan Y, Wu G, Wang S, Yuan L (2019). NEK7 interacts with NLRP3 to modulate the pyroptosis in inflammatory bowel disease via NF-κB signaling. Cell Death Dis.

[43] Zhang T, Ding C, Chen H, Zhao J, Chen Z, Chen B (2022). m(6)A mRNA modification maintains colonic epithelial cell homeostasis via NF-κB-mediated antiapoptotic pathway. Science Adv.

