# Supplementary figures and images for: Muc2 mucin o-glycosylation interacts with enteropathogenic Escherichia coli to influence the development of ulcerative colitis based on the NF-kB signaling pathway
Source: J Transl Med. 2023 Nov 8;21:793. doi: 10.1186/s12967-023-04687-2 (PMC10631195; doi:10.1186/s12967-023-04687-2)

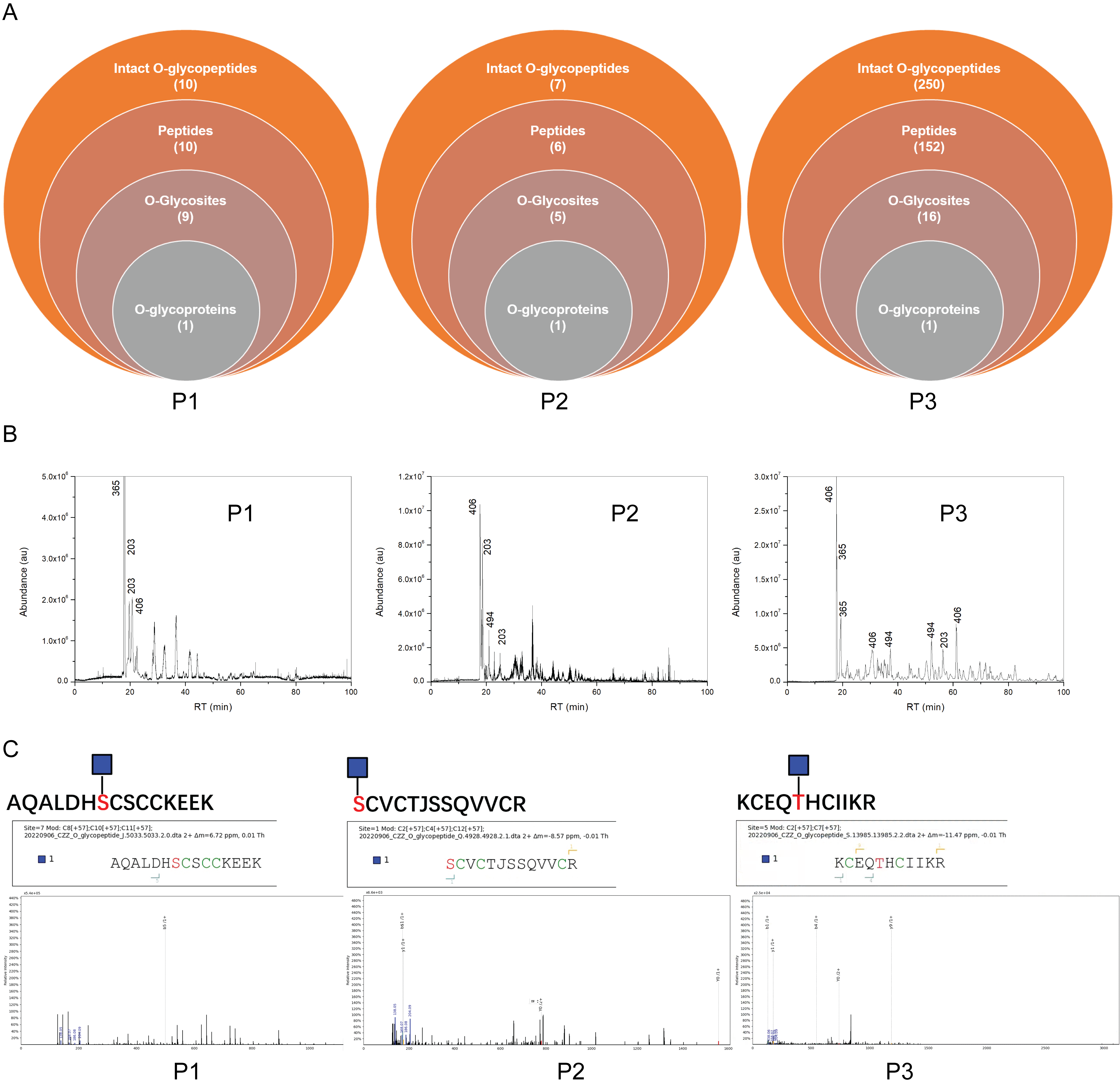

Supplement: Supplementary file 1 — Additional file 1: Figure S1. Identification of intact O-glycopeptides in intestinal tissues obtained from three additional patients with UC. (A) Venn diagram illustrating the presence of intact O-glycopeptides in tissues samples of patients with UC. (B) The abundance of O-glycans in tissue samples of patients with UC. (C) The binding of intact O-glycopeptides to MUC2 protein in tissue samples of patients with UC. P represents tissues obtained from patients with UC. [file 12967_2023_4687_MOESM1_ESM.tif]

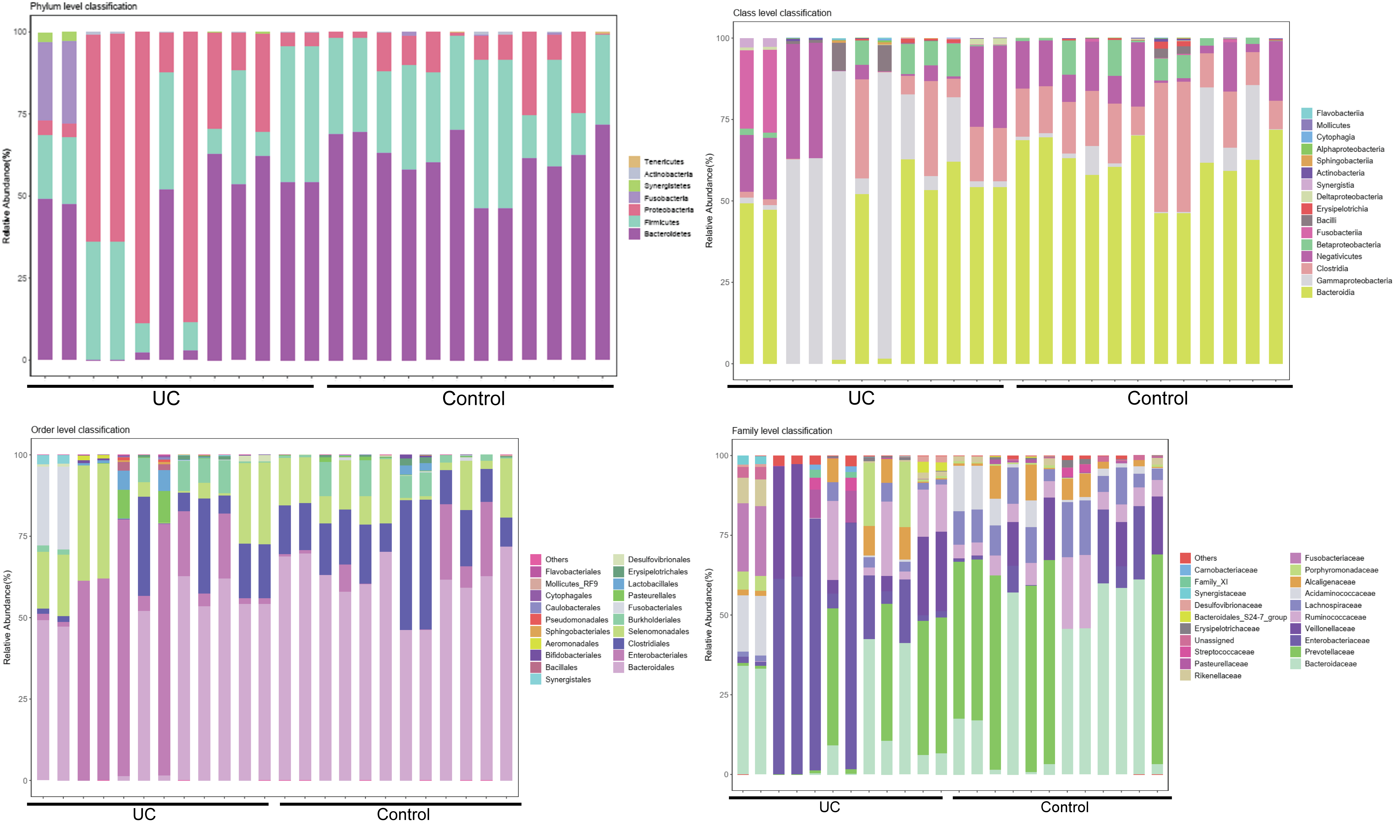

Supplement: Supplementary file 2 — Additional file 2: Figure S2. The relative abundance of fecal microbiota identified at the phylum, class, order, and family levels. [file 12967_2023_4687_MOESM2_ESM.tif]

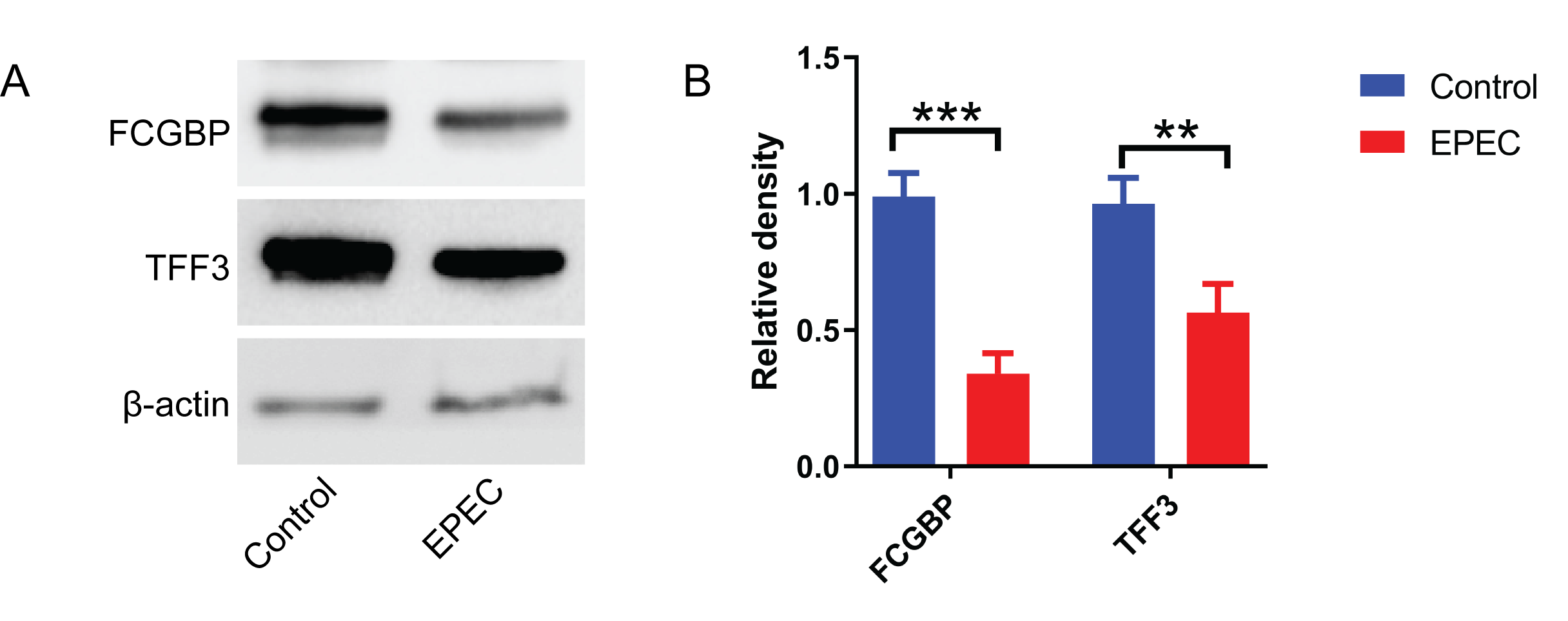

Supplement: Supplementary file 3 — Additional file 3: Figure S3. The protein levels of TFF3 and FCGBP in intestinal tissues of EPEC-treated mice. Differences between two groups were assessed using Student’s t-test. **P< 0.01; ***P< 0.001. [file 12967_2023_4687_MOESM3_ESM.tif]

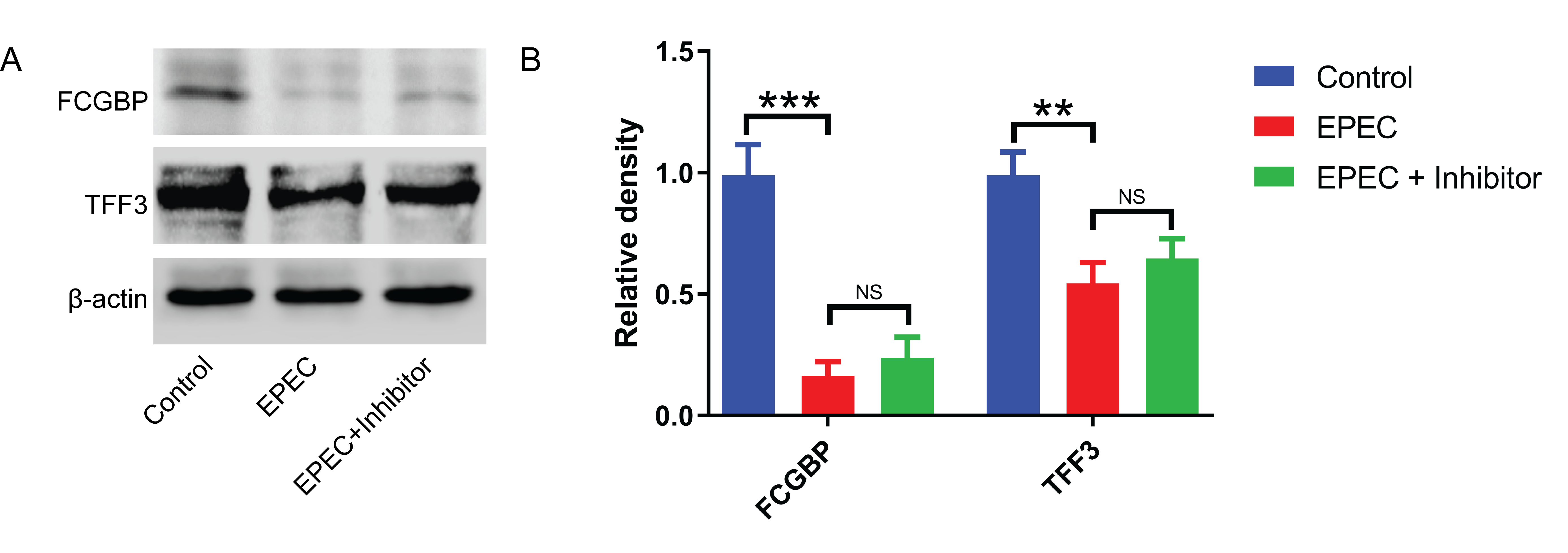

Supplement: Supplementary file 4 — Additional file 4: Figure S4. The protein levels of TFF3 and FCGBP in intestinal tissues of EPEC E. coli and benzyl-α-GalNAc cotreated mice. Differences among the multiple groups were analyzed by one-way ANOVA. *P< 0.05; **P< 0.01; ***P< 0.001. [file 12967_2023_4687_MOESM4_ESM.tif]
